# Risk of Malignancy and Tuberculosis of Biological and Targeted Drug in Patients With Spondyloarthritis: Systematic Review and Meta-analysis of Randomized Controlled Trials

**DOI:** 10.3389/fphar.2021.705669

**Published:** 2021-10-29

**Authors:** Siliang Man, Lidong Hu, Xiaojian Ji, Yiwen Wang, Yingpei Ma, Lei Wang, Jian Zhu, Feng Huang

**Affiliations:** ^1^ Department of Rheumatology, Beijing Jishuitan Hospital, Beijing, China; ^2^ School of Medicine, Xiamen University, Xiamen, China; ^3^ Department of Rheumatology and Immunology, the First Medical Centre, Chinese PLA General Hospital, Beijing, China

**Keywords:** spondyloarthritis, biologic therapy, malignancy, tuberculosis, systematic review and meta-analysis

## Abstract

**Objective:** Concerns exist regarding the potential development of malignancy and tuberculosis in patients with spondyloarthritis (SpA) treated with biologics. We assessed the extent to which biologic therapy may increase the risk of malignancy and tuberculosis in patients with SpA by meta-analysis to derive estimates of sparse harmful events occurring in Randomized Controlled Trials (RCTs).

**Methods:** A systematic literature search was conducted in PubMed, EMbase, Web of Science, the Cochrane Library, and China Biology Medicine disc for RCTs evaluating the risk of sparse harmful events of biologic therapy in patients with SpA from inception through August 9, 2021. We calculated a pooled Peto OR for malignancy and tuberculosis in biologics-treated patients vs. placebo patients. The risk of bias on the included RCTs was assessed by using Cochrane Risk of Bias tool.

**Results:** In total, 63 studies were included in this meta-analysis, and 83 patients and 7 patients developed malignancy and tuberculosis, respectively. Overall, the risk of malignancy and tuberculosis was increased in SpA patients treated with biologics compared to placebo (malignancy: Peto OR: 2.49, 95%CI: 1.61–3.87, *p* < 0.001; tuberculosis: Peto OR: 5.98, 95%CI: 1.29–27.76, *p* = 0.022). Remarkably, compared to placebo, there was higher risk of malignancy for IL-17 inhibitors (Peto OR: 3.68, 95%CI: 1.20–11.30, *p* = 0.023) and small molecule targeted drugs (Peto OR: 3.08, 95%CI: 1.37–6.90, *p* = 0.043) in peripheral SpA, and for TNF receptor-Fc fusion protein in axial SpA (Peto OR: 7.18, 95%CI: 1.21–42.69, *p* = 0.030). Besides, the risk of tuberculosis was higher for anti-TNFα antibody in axial SpA (Peto OR: 6.17, 95%CI: 1.03–37.13, *p* = 0.046).

**Conclusion:** This meta-analysis showed an elevated risk of malignancy in patients with peripheral SpA treated with biologics, especially for IL-17 inhibitors, and small molecule targeted drugs, a slightly increased risk of malignancy in TNF receptor-Fc fusion protein in axial SpA, and increased risk of tuberculosis in patients with axial SpA treated with anti-TNFα antibody. These findings need to be validated by studies with larger population and longer follow-up.

## Introduction

Spondyloarthritis (SpA) is a series of chronic inflammatory conditions that have a range of manifestations, including predominantly axial SpA (radiographic axial SpA (axSpA) and non-radiographic axial SpA (non-axSpA)) and peripheral SpA (enteropathic arthritis, reactive arthritis, and psoriatic arthritis). People with predominantly axSpA may have additional peripheral symptoms, and vice versa. Treatment with non-steroidal anti-inflammatory drugs (NSAIDs) or conventional synthetic disease-modifying anti-rheumatic drugs (csDMARDs), or a combination of both, can usually ameliorate disease activity and retard joint damage, thereby improving quality of life of patients with SpA. However, in a sizeable proportion of patients with SpA, NSAIDs or csDMARDs fail or are not tolerated. For these patients not responding to NSAIDs or csDMARDs, biologics or small molecular targeted drugs can provide clinically important improvement via targeting specific inflammatory mediators in inflammatory pathways, alleviating inflammation, and thus better controlling symptoms and structural destruction. Concerns have been raised about the safety of biologics or small molecular targeted drugs, especially with regard to malignancies and tuberculosis, because they can interfere with the immune system.

Cancer is a common event in individuals with rheumatic diseases. A study suggested that about 20% of individuals with rheumatoid arthritis will be diagnosed with malignancy during their remaining lifetimes (Cush and Dao, 2012). When analyzing this risk, some confounding factors have to be taken into account. For example, treatments for the rheumatic diseases that act on the immune system, such as biological agents, may play a significant role in favoring cancer. At present, most of the researches on tumorigenicity of biologics are on TNF-α inhibitors for the treatment of rheumatoid arthritis, but few are in SpA. The use of biologics was not significantly associated with an increased risk of malignancy in individuals with rheumatoid arthritis who were included in randomized controlled trials (RCTs) for at least 6-months duration, compared with other csDMARDs or with placebo (Lopez-Olivo et al., 2012). Because of the different pathogenesis between rheumatoid arthritis and SpA, it is necessary to investigate the occurrence of malignancy in the treatment of SpA with biologics.

Tuberculosis is an important infectious disease worldwide, which is related to a significant morbidity and mortality, especially in developing countries. More and more biologics are used in the treatment of individuals with SpA, which is a matter of great concern, particularly for the individuals having previously suffered from tuberculosis. Studies in Taiwan have shown a 2.28-fold increase in the risk of tuberculosis in patients with rheumatoid arthritis than that in the general population (Liao et al., 2015), while patients treated with TNF-α inhibitors were at higher risk of developing tuberculosis than those treated with other medications (Lim et al., 2016). Hence, the link between biological agents and the occurrence of tuberculosis is certainly an area of concern.

At present, many RCTs have reported the safety of biologics and small molecular targeted drugs in the treatment of SpA. However, RCTs are inadequate for detecting and quantifying rare events, such as malignancies and tuberculosis. A meta-analytic approach is considered useful to overcome the inherent limitations of individual RCTs in the assessment of safety outcomes. The main objective of the systematic review is to summarize and contextualize the risk of malignancies and tuberculosis accompanying biologics and small molecular targeted drugs use in RCTs and long-term extension studies using meta-analysis.

## Materials and Methods

We strictly followed PRISMA (the Preferred Reported Items for Systematic Reviews and Meta-analyses) guidelines and the recommendations from the Cochrane Collaboration to conduct this systematic review and meta-analysis.

### Data Sources and Searches

An experienced medical librarian and information specialist was invited to conduct a comprehensive literature search with input from the study team. The following electronic bibliographic databases: PubMed, EMbase, the Cochrane Library, Web of Science, and China Biology Medicine disc (CBM), were searched from inception through August 9, 2021. No limits were applied to race, sex, or language, except for human subjects. The details of search strategies for electronic database were showed in Supplementary Appendix A1. Other resources were hand-searched, including Websites and bibliographic references from systematic reviews and RCTs of interest, for additional citations not identified through the original search strategy.

### Selection of the Trials

Study inclusion was assessed by two pairs of independent reviewers (SM and XJ, LW and YW). Disagreements were discussed and resolved by consensus and, when needed, a third reviewer acted as an adjudicator (LH) until a consensus was reached.

### Eligible Trials Were Required to

Type of study design: Randomized controlled trials (RCTs) that have been published in peer-review journals and had at least one 24-weeks follow-up.

Type of patients: Patients (≥18 years old) with SpA including axial SpA and peripheral SpA confirmed by physician/specialist according to the 1984 modified New York criteria and ASAS classification criteria for SpA.

Type of intervention and comparator: Studies comparing any biologics (TNF-α inhibitors, IL-17 inhibitors, IL-6 inhibitors, IL-23 inhibitors, and small molecule targeted drugs and so on) against non-biologics (placebo, NSAIDs or csDMARDs).

Outcomes: Reporting at least one outcome of malignancy or tuberculosis.

### Data Extraction and Risk of Bias Assessment

Two reviewers (SM and XJ) independently individual trial data, and two additional reviewers (LW and YW) cross-checked the extracted results. Disagreements were discussed and solved through consensus, and a third reviewer acted as an adjudicator (LH) if necessary. From each selected trial, we collected general information (e.g. authors’ name, publication year, country, and study design), study population (e.g. age of patients, gender distribution), and intervention characteristics (details of intervention and control, duration of intervention and follow-up). Primary outcome data was number and type of malignancies and tuberculosis.

All included trials were assessed for risk of bias by two reviewers (SM and XJ) with version 2 of the Cochrane Risk of Bias Assessment tool. The following domains of individual trials were assessed: random sequence generation, allocation concealment, blinding, incomplete outcome data, selective outcome reporting, and other biases (including carryover, extreme baseline imbalance, and funding). We assessed the risk of bias using the categories of yes (low risk of bias), no (high risk of bias), and unclear (lack of information or uncertainty about the potential for bias).

### Data Synthesis and Analysis

We identified the number of patients with at least one malignancy or tuberculosis, based on the analysis of adverse event in individual trial. The number of patients with SpA receiving at least one dose of study drug represented the denominator of our outcome measurement.

Our study protocol required the use of a fixed-effect model for meta-analysis due to its superior performance while pooling the clinical trials with few or rare events, comparing to random-effect model, and the results were expressed as Peto odds ratio (OR) and associated 95%CI. Peto OR is preferred for few or rare events, and it is not necessary to have corrections for zero cell counts in a single group. We stratified by biologics classes to explore how different biologics classes affect the risk of malignancy and tuberculosis infection. Since there were fewer cases of malignancy and tuberculosis, we could not compare the dose or frequency of administration. Therefore, for the clinical trials that investigated different doses and frequencies of the same biologics class, we pooled cases of malignancy or tuberculosis across the different doses and frequencies for analysis. The I (Lopez-Olivo et al., 2012) statistic was used to assess heterogeneity across RCTs. If the I (Lopez-Olivo et al., 2012) value was over 50%, substantial heterogeneity was present, and then the possible causes needed be investigated.

Meta-analysis was conducted using “meta” package on R 3.6.2 software. *p*-value < 0.05 was considered as statistically significant in all tests.

## Results

### Literature Selection and Trial Characteristics

A total of 13,103 unique citations were identified through electronic bibliographic databases and hand-searching. There were 8,925 records that were potentially relevant to our topic in our first selection round, of which 166 were deemed eligible for full review. Finally, a total of 63 trials met inclusion criteria for our systematic review and meta-analysis. All the included studies were reported in Chinese or English. The details of study selection process were showed in Supplementary Figure S1.

The 63 RCTs containing 19,291 patients with SpA, were published between 2003 and 2021. Among these RCTs, 27 investigated axSpA and 36 investigated peripheral SpA. A majority of RCTs were two-arms clinical trials, with follow-up periods ranging from 12 to 144 weeks (Supplementary Table S1).

### Risk of Bias Assessments

**FIGURE 1:**
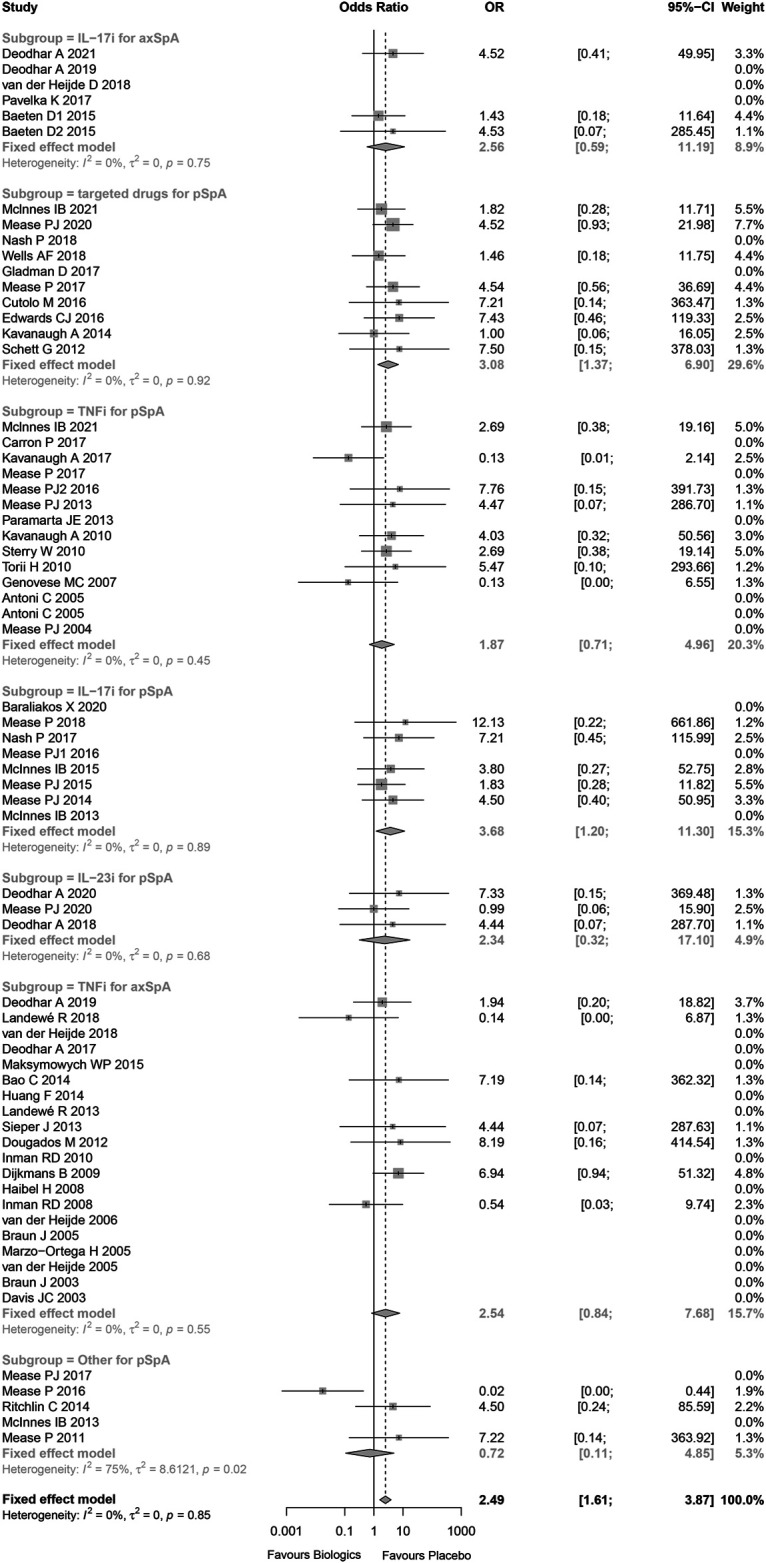
Risk of bias graph.

All studies provided sufficient details of randomization. Although most of the clinical trials declared that they were double-blind, more than half of the trials indicated inadequate in the method of allocation concealment. In all studies, the co-interventions and baseline characteristics were similar between the biologics group and control group, and control groups (placebo and) were grouped together. In some patients who previously received placebo or csDMARDs, switching from place to active medication might introduce a potential risk of bias. In some trials (28/58), the method of imputation of no response, with “advancement penalty”, was used to address this potential risk of bias. The risk of bias graph of assessment for all the included RCTs was demonstrated in Figure 1. Risk of bias summary was shown in Supplementary Figure S2.

### Malignancy

Published data in 63 retrieved RCTs reported 69 malignancies in 11,281 patients receiving at least one dose of biologics (0.6%) and 14 malignancies in 7,913 control patients (0.2%). Of the 83 malignancies, 55 were solid tumors (ie, prostate cancer, gastric adenocarcinoma), 17 were skin cancer (ie, melanomia, squamous cell cancer, and basal cell cancer), 4 were lymphomas, and 7 were not specified. Mean durations of placebo exposure and active treatment exposure were 21 ± 9 weeks and 37 ± 22 weeks. Malignancies occurred in 12, 29, 27, and 7 patients with durations of active treatment exposure more than 52 weeks, 24–52 weeks, less than 24 weeks and less than 12 weeks, respectively.

**FIGURE 2:**
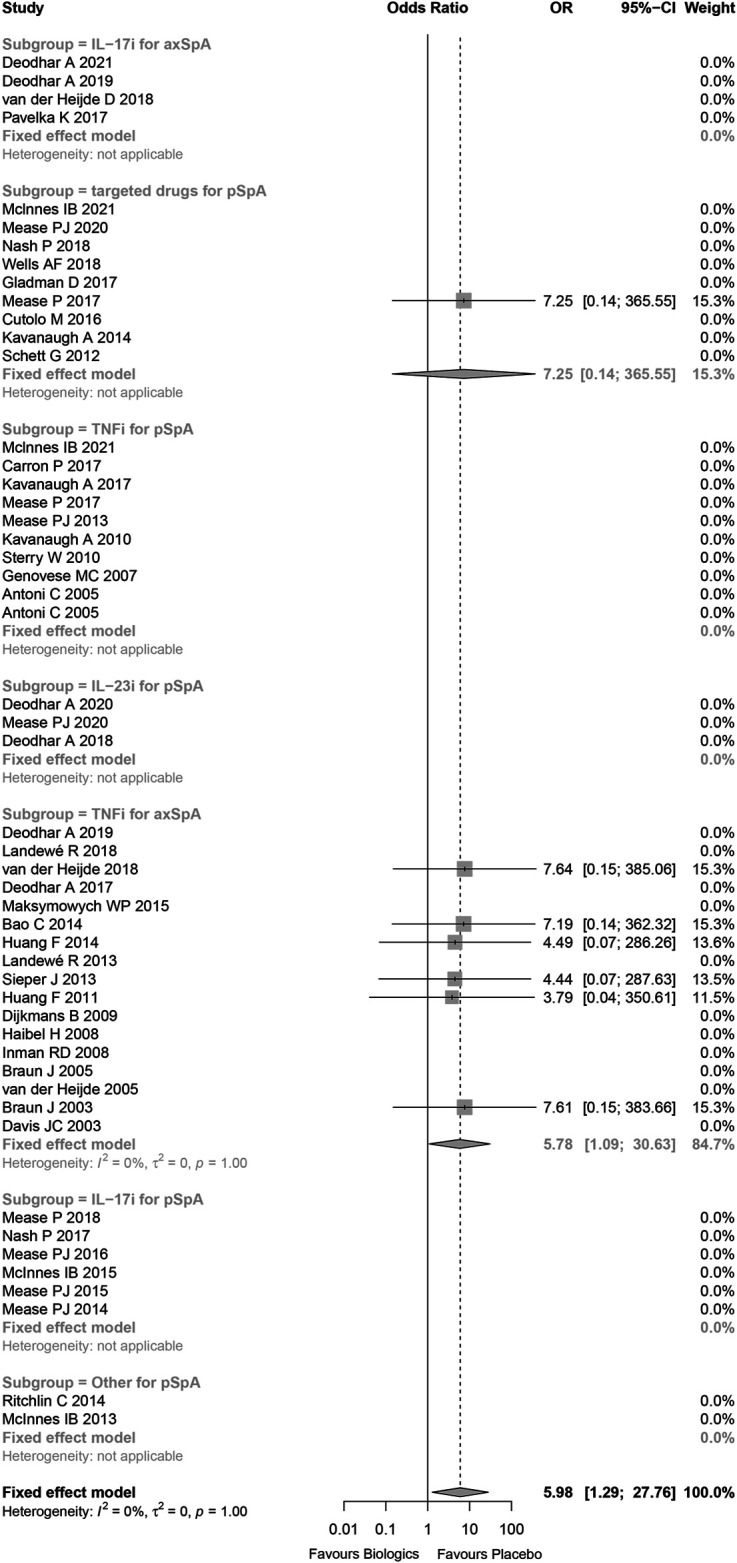
Forest plot of meta-analyses comparing biologics versus placebo for risk of malignancy.

The measurement of inconsistency between the RCTs (I (Lopez-Olivo et al., 2012)) was 0% (*p* = 0.85), which indicated that there was no statistical heterogeneity in these trials. Overall, there was an increased risk of malignancies in individuals with SpA using biological and targeted drugs vs. placebo patients (Peto OR 2.49, 95%CI 1.61–3.87, *p* < 0.001; Figure 2). The overall statistical significance did not change, while omitting any single trials, which indicated that the results were statistically robust (Supplementary Figure S3).

Subgroup analysis of trials was conducted by using biologics class. The risks of malignancy were higher than placebo for IL-17 inhibitors in patients with peripheral SpA (Peto OR 3.68, 95%CI 1.10–11.30, *p* = 0.023; I^2^ = 0%, and *p* = 0.889) without evidence of publication bias (Supplementary Figure S4), small molecule targeted drugs in patients with peripheral SpA (Peto OR 3.08, 95%CI 1.37–6.90, *p* = 0.006; I^2^ = 0%, and *p* = 0.923) without evidence of publication bias (Supplementary Figure S5). In addition, while omitting these studies being high-risk bias, the results still indicated that the risks of malignancy were higher than placebo for IL-17 inhibitors in patients with peripheral SpA, small molecule targeted drugs in patients with peripheral SpA (Supplementary Figure S6).

There was no significant difference between TNF-α inhibitors and placebo in patients with axial SpA (Peto OR 2.54, 95%CI 0.84–7.68, *p* = 0.099; I^2^ = 0%, and *p* = 0.551) without evidence of publication bias (Supplementary Figure S7). A subgroup analysis was performed based on anti-TNFα antibody and TNF receptor-Fc fusion protein. The results showed that the risk of malignancy was higher than placebo for TNF receptor-Fc fusion protein in patients with axial SpA (Peto OR 7.18, 95%CI 1.21–42.69, *p* = 0.030; I^2^ = 0%, and *p* = 0.940), while anti-TNFα antibody was not (Peto OR 1.32, 95%CI 0.32–5.43, *p* = 0.700) (Supplementary Figure S8).

Compared to placebo, there were slightly increased risk of malignancy for IL-23 inhibitors in patients with peripheral SpA (Peo OR 2.34, 95%CI 0.32–17.10, *p* = 0.403; I^2^ = 0%, and *p* = 0.676), IL-17 inhibitors in patients with axial SpA (Peto OR 2.56, 95%CI 0.59–11.19, *p* = 0.212; I^2^ = 0%, and *p* = 0.750) without evidence of publication bias (Supplementary Figure S9), and TNF-α inhibitors in patients with peripheral SpA (Peto OR 1.87, 95%CI 0.71–4.96, *p* = 0.206; I^2^ = 0%, and *p* = 0.450) without evidence of publication bias (Supplementary Figure S10–11). Nevertheless, all Peto ORs had wide 95%CI with *p* > 0.05, that was, there were no statistical difference between the treatment groups and placebo.

### Tuberculosis

Tuberculosis infections were reported in 7 patients in biological and targeted drugs therapy group, but no tuberculosis was reported in placebo group. Five of them occurred in areas with relatively higher background tuberculosis rates (China, Mexico, and Russia), and the other two in Germany. Two cases of tuberculosis occurred in the patients treated with adalimumab, two in patients treated with infliximab, one in patients treated with golimumab, and one in patients treated with tofacitinib.

**FIGURE 3:**
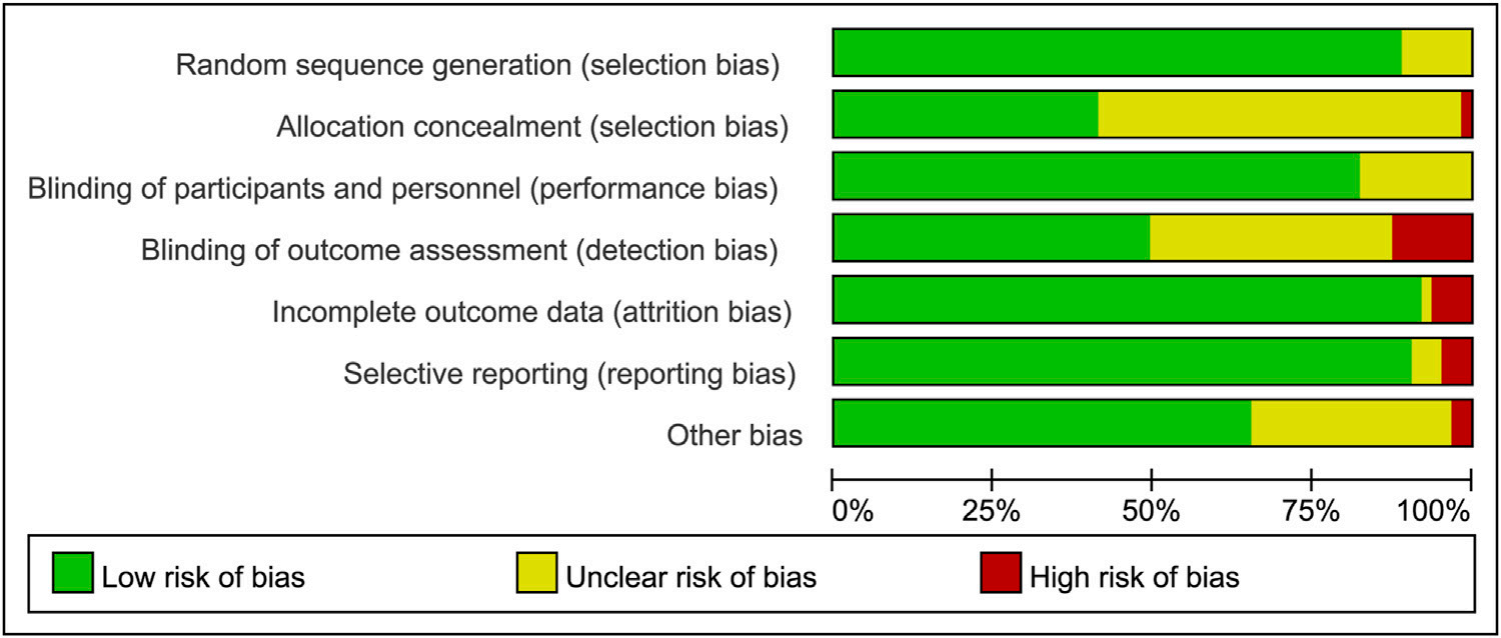
Forest plot of meta-analyses comparing biologics versus placebo for risk of tuberculosis

The statistical heterogeneity was very low (I (Lopez-Olivo et al., 2012) = 0%) and not beyond variations that might be caused by chance (*p* = 1.00). Comparing to placebo, the risk of tuberculosis infection in patients treated with biologics or targeted drugs was increased (Peto OR 5.98, 95%CI 1.29–27.76, *p* = 0.022; Figure 3) without evidence of publication bias (Supplementary Figure S12). 71.4% of tuberculosis infections occurred in anti-TNFα antibody for axial SpA group, and the result demonstrated that there was significant increase in anti-TNFα antibody for axial SpA (Peto OR 6.17, 95%CI 1.03–37.13, *p* = 0.046; I^2^ = 0%, *p* = 0.999) without evidence of publication bias (Supplementary Figure S13). No tuberculosis infectious was found in TNF receptor-Fc fusion protein (Supplementary Figure S14). In addition, no tuberculosis occurred in the studies being high-risk bias, so there was no effect on the above results while omitting these studies being high-risk bias (Supplementary Figure S15).

## Discussion

There has been concern regarding a putative increasing risk of malignancies and tuberculosis with either biologics and small molecular targeted drugs treatment because of the impacts of these therapies on the immune system. Our work shows that there is an elevated risk of malignancies and tuberculosis in patients with SpA receiving biologics or small molecular targeted drugs therapy, compared to placebo. While investigating the risk of malignancies in the treatment with biologics by different types of biological agents in each SpA type, we find a wide 95%CI and *p* > 0.05 in all pooled Peto ORs, except for IL-17 inhibitors and small molecular targeted drugs in peripheral SpA, compared to placebo. In addition, we only find an elevated risk of tuberculosis infection in individuals with axial SpA receiving TNF-ɑ inhibitors therapy.

### Malignancies

Malignancies in single RCTs of biological and targeted drugs treatment in patients with SpA were rare, and the observed differences were not statistically significant in their occurrence between groups. We investigated the risk of malignancies in treatment with biologics and small molecular targeted drugs in SpA including axial SpA and peripheral SpA by meta-analysis, overall as well as by biological agents’ classes in each SpA type.

The comparison of IL-17 inhibitors to placebo was the only one biologic that achieved significant difference in patients with peripheral SpA rather than axial SpA. Nevertheless, all seven included RCTs investigated exclusively individuals with psoriatic arthritis (a specific subtype of peripheral SpA). Interestingly, a comprehensive clinical trial safety dataset consisting of twenty-three RCTs of secukinumab in ankylosing spondylitis, psoriatic arthritis, and psoriasis indications indicated that the exposure-adjusted incident rates per 100 patient-years for malignancies in treatment with secukinumab were 0.8, 1.1, and 0.5 in the psoriasis, psoriatic arthritis, and ankylosing spondylitis studies, respectively (Deodhar et al., 2019). Because of the double-edged nature of IL-17, its exact role in tumorigenesis and metastasis is still unclear (Chang, 2019). On the one hand, IL-17 can play a tumor-promoting effect by inducing tumor angiogenesis, which increases the risk malignancies. On the other hand, IL-17 can mediate anti-immunity by enhancing the activity of natural killer cells and cytotoxic T lymphocytes, which decreases the risk of malignancies. There are reasons to believe that any increased risk for malignancy may be contributed in part by the nature of chronic inflammation on the disease with the involvement of IL-17. We recommend a long-term follow-up of these patients to investigate whether the tumor occurs or not and further research to elucidate the differential tumorigenic effects of IL-17 inhibitors in treatment of psoriatic arthritis and ankylosing spondylitis.

The comparison of TNF-α inhibitors to placebo in SpA including peripheral SpA and axial SpA did not achieve statistical significance. A collaborative study from the ARTIS and DANBIO registers showed that treatment with TNF-α inhibitors in patients (n = 8703) with SpA was not associated with increased risks of cancer, neither overall nor for the most cancer types, such as prostate cancer, lung cancer, colorectal cancer, lymphoma, breast cancer, and malignant melanoma (Hellgren et al., 2017). A subgroup analysis was performed based on anti-TNFα antibody and TNF receptor-Fc fusion protein in our study. The results showed that the risk of malignancy was higher than placebo for TNF receptor-Fc fusion protein in patients with axial SpA, while anti-TNFα antibody was not. However, only two studies reported the cases of malignancy, and pooled 95%CI was wide and imprecise, which may require more high-quality clinical trials to verify this result and also prompts us to pay attention to the effect of TNF receptor-Fc fusion protein on malignancy. As for anti-TNFα antibody, many studies indicated that it did not increase the risk of malignancy, which was consistent with our findings. A study by Burmester GR et al. analyzed the long-term safety of adalimumab treatment (Burmester et al., 2013). This analysis included 1,684 patients exposed to adalimumab in 4 clinical trials in ankylosing spondylitis, and results showed that standardized incidence rate for all malignancies including lymphoma and non-melanoma skin cancer was similar to the general population.

Additionally, in our study, there was a statistically significant increase in the risk of malignancies accompanying small molecule targeted drugs use, relative to placebo. In our study, two clinical trials on tofacitinib reported the occurrence of malignancy, one of which showed no tumorigenesis, and the other showed four patients with malignancy in the tofacitinib group. Besides, there were 8 patients with malignancy in the apremilast group, while only 2 patients with malignancy in placebo group. Due to the limitations of the shorter observation period in RCTs and the fact that these small molecule targeted drugs are newly used to treat SpA, our findings accentuate the necessity for long-term observational studies.

### Tuberculosis

In our study, the main finding was that anti-TNFα antibody significantly increased the risk of tuberculosis infection in individuals with axial SpA. The increasing risk of tuberculosis is a main safety issue for anti-TNF-α therapy. Three widely used anti-TNF-α drugs with long-term safety profiles-infliximab and adalimumab-have been indicated to increase the risk of developing tuberculosis, which is similar to our finding. Anti-TNF-α therapies are well known to have a significant effect on immune cells, deactivating T cells and macrophages, and induction of apoptosis in key immune cells (Mitoma et al., 2008; Harris and Keane, 2010). Moreover, this has also been confirmed in the mouse models where TNF-α gene-deficient mice were infected with tuberculosis pathogens to study the role of TNF-α in tuberculosis infection (Kindler et al., 1989; Ehlers et al., 1999). The differences in the risk of tuberculosis infection associated with different anti-TNF-α drugs may be attributed to subtle differences in their mechanism of action. Monoclonal antibodies can increase the risk of tuberculosis infection by inhibiting the activation of T cells and the release of interferon-γ (Taylor, 2010). Due to different mechanisms of action, anti-TNF-α drugs, such as adalimumab, infliximab, and etanercept, are often studied and compared. In a national study in South Korea, it was found that the incidence of tuberculosis infection was highest among patients receiving infliximab therapy (IRR: 6.8), followed by adalimumab (IRR: 3.5) and etanercept (Jung et al., 2015). A study in the United States also showed that etanercept and infliximab were associated with tuberculosis infection, and the reporting rate of tuberculosis infection in patients using etanercept was lower than that of those using infliximab (10 cases vs. 41 cases per 100,000 patient-years of exposure) (Mohan et al., 2004). The standard of care in SpA adhere to the statement on the indications and safety monitoring for anti-TNF-α therapy, and advocate to assess patients for active and latent tuberculosis prior to treatment initiation (Kim et al., 2020). A study reported that the rate of active tuberculosis in adalimumab clinical trials has decreased from 15/1,000 patient-years to 2/1,000 patient-years, due to the implementation of screening and prophylaxis of latent tuberculosis infection in 1998 and 1999, respectively (Kim et al., 2020).

## Limitations

There are several study limitations to consider. First, the short period of exposure in the included RCTs is a major solid limitation. Therefore, our results can only represent short- or medium-term risk assessment of malignancy and TB using biologics. Second, definitions of malignancies and TB may differ by individual studies, and many studies did not strictly follow the standard definition of tuberculosis and malignancies within the Medical Dictionary for Regulatory Activities (MeDRA) dictionary. These cases were collected based on the original publication case description only with relevant differences between studies. These may result in a bias in collecting these cases. Third, there may be a bias for safety evaluation due to the patients previously exposed to biologics. Of the included studies, 24 studies explicitly excluded the patients previously treated with any biologics, 19 studies allowed the patients prior to biologics efficacy failures, but this was generally limited to ≤30% of enrolled patients, and the other studies did not report this information. Since the detailed information on individuals previously exposed to biologics were not reported, it is difficult to perform further subgroup analysis. Fourth, for *in situ* malignancies, in some countries, screening may be mandatory or highly recommended. However, in all the included studies, malignancies were only reported as an adverse event, and the details of the screening for *in situ* malignancies were not disclosed. It should also be noted that malignancies that occur within the short period of RCTs may not be new onset. It is necessary to perform in-depth screening while recruiting patients, due to the contraindications of biologics. Fifth, the doses of frequency of a biologic varied greatly among the included studies. It is difficult to performed a sensitivity analysis by dose. Therefore, this may be a bias for safety evaluation. Sixth, region of study performance and standard of care may differ by region on top of the local incidence of tuberculosis. However, it is difficult to compare the difference between studies conducted in regions where the background risk of tuberculosis may vary, as only six cases of tuberculosis were reported in the included studies. Seventh, screening and care for tuberculosis may change a lot over time (2003–2020). However, screening for tuberculosis were reported in only 11 of the 58 clinical trials, and were mostly based on PPD, chest radiographs, IGRA, or a combination, and the detailed information of screening, diagnosis and standard of care for tuberculosis were unavailable to us. This may result in a difference in the number of reported tuberculosis cases. Eighth, we only included these RCTs that have been published in peer-review journals since the results of these studies is more reliable. However, this may also lead to the potential publication bias. We have discussed them in the Limitation.

Above all, this systematic review and meta-analysis showed an increased risk of malignancies in individuals with peripheral SpA receiving biologics therapy, particularly for IL-17 inhibitors, and small molecule targeted drugs, a slightly increased risk of malignancy in TNF receptor-Fc fusion protein in axial SpA, and increased risk of tuberculosis in individuals with axial SpA treated with anti-TNFα antibody. Our findings may have direct implications in the management of a large number of patients treated currently with biologics and small molecule targeted drugs. In addition, due to limitations of very small number of events of malignancy and tuberculosis, our findings need to be validated by studies with larger population and longer follow-up.

## Data Availability

The original contributions presented in the study are included in the article/Supplementary Material, further inquiries can be directed to the corresponding author.
