# Supplementary figures and images for: Risk of Malignancy and Tuberculosis of Biological and Targeted Drug in Patients With Spondyloarthritis: Systematic Review and Meta-analysis of Randomized Controlled Trials
Source: Front Pharmacol. 2021 Oct 29;12:705669. doi: 10.3389/fphar.2021.705669 (PMC8585981; doi:10.3389/fphar.2021.705669)

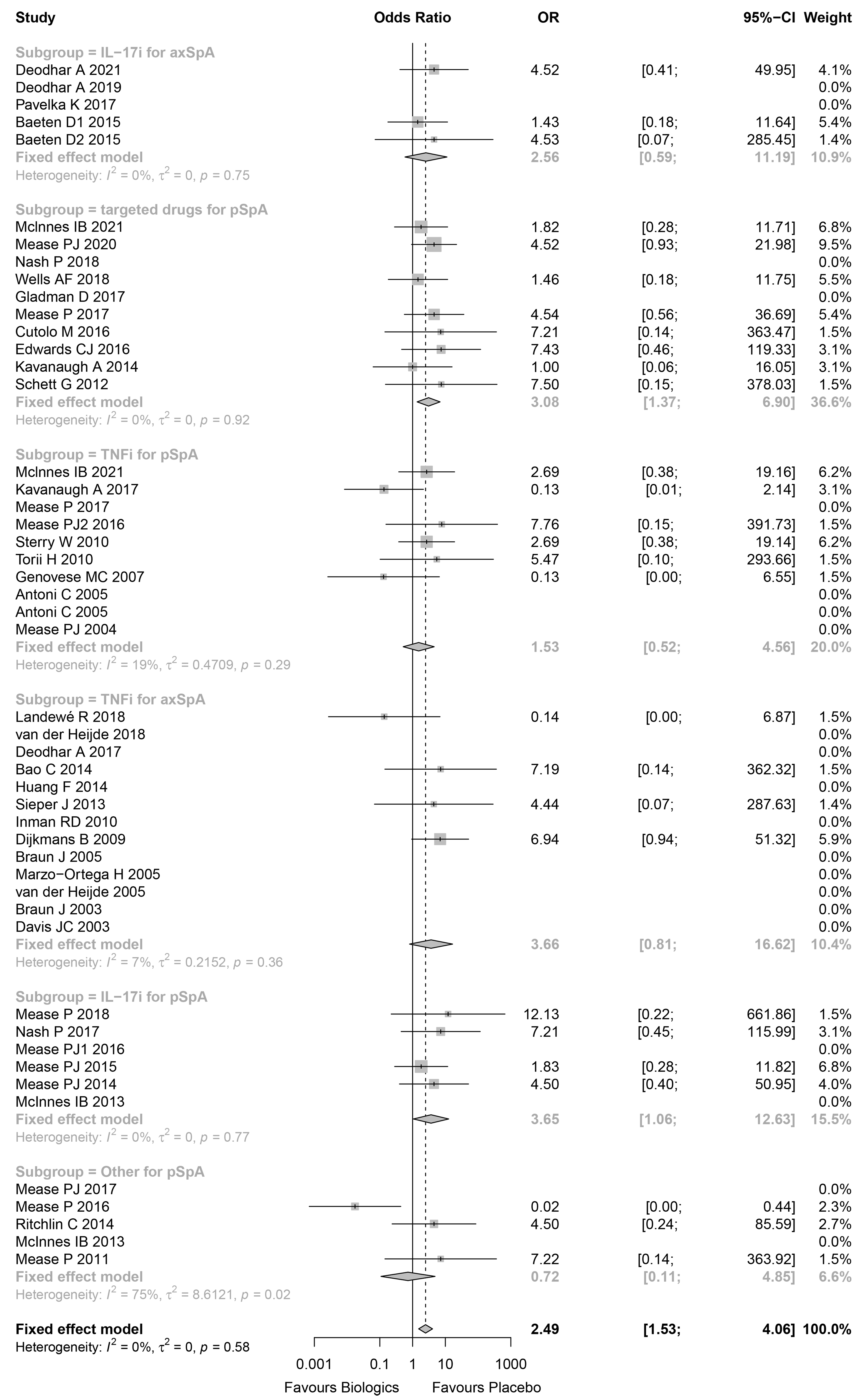

Supplement: Supplementary file 1 [file Image6.TIF]

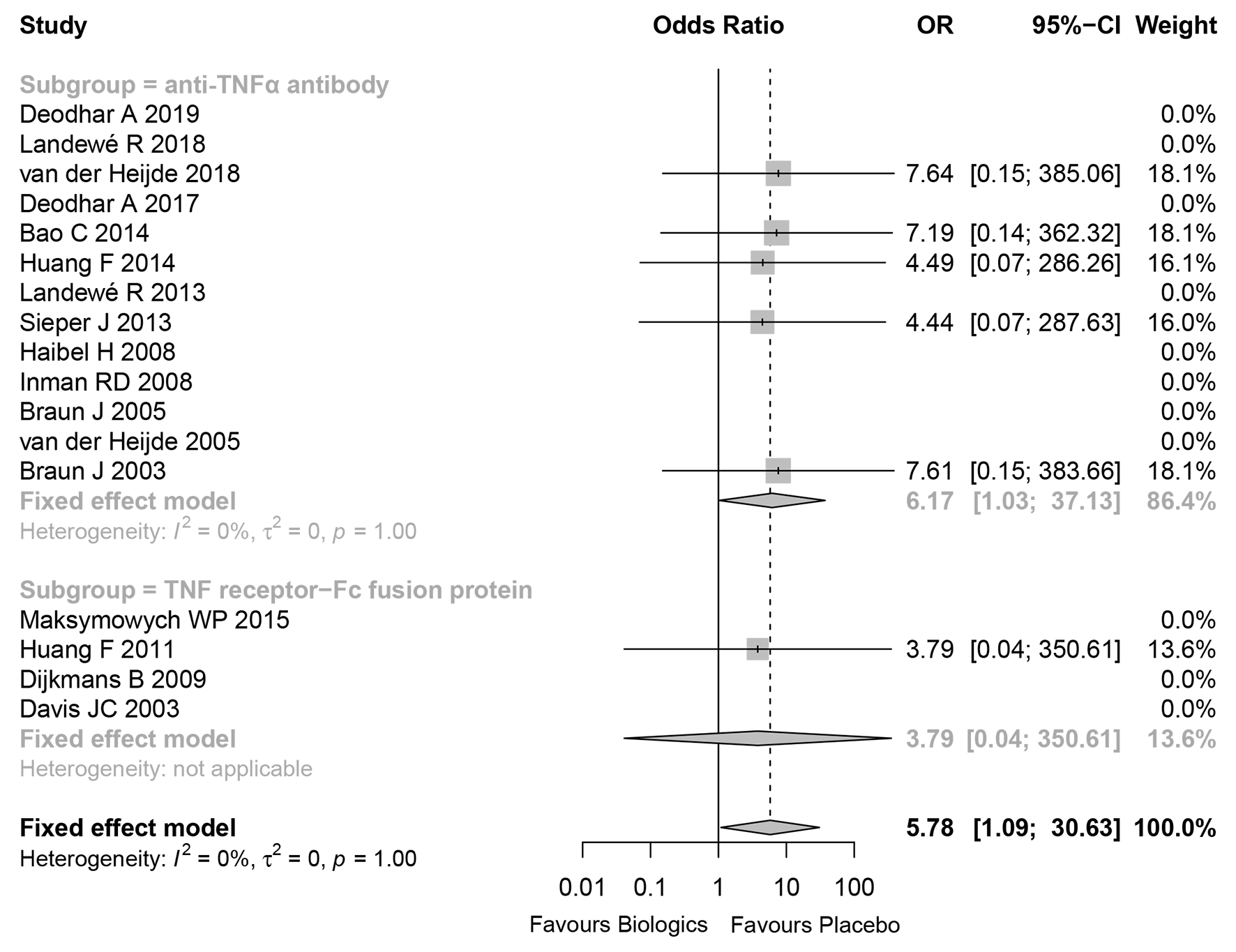

Supplement: Supplementary file 2 [file Image14.TIF]

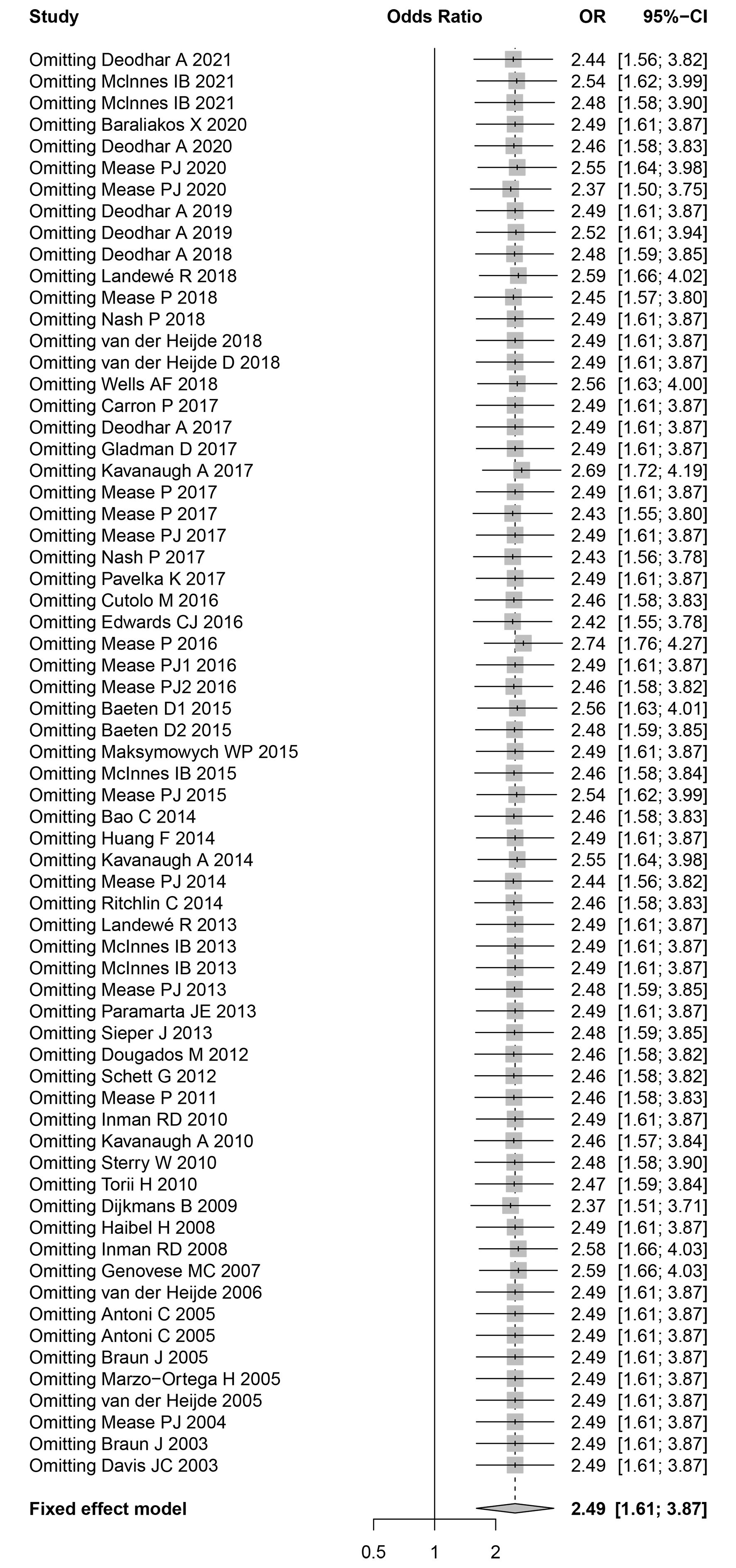

Supplement: Supplementary file 3 [file Image3.TIF]

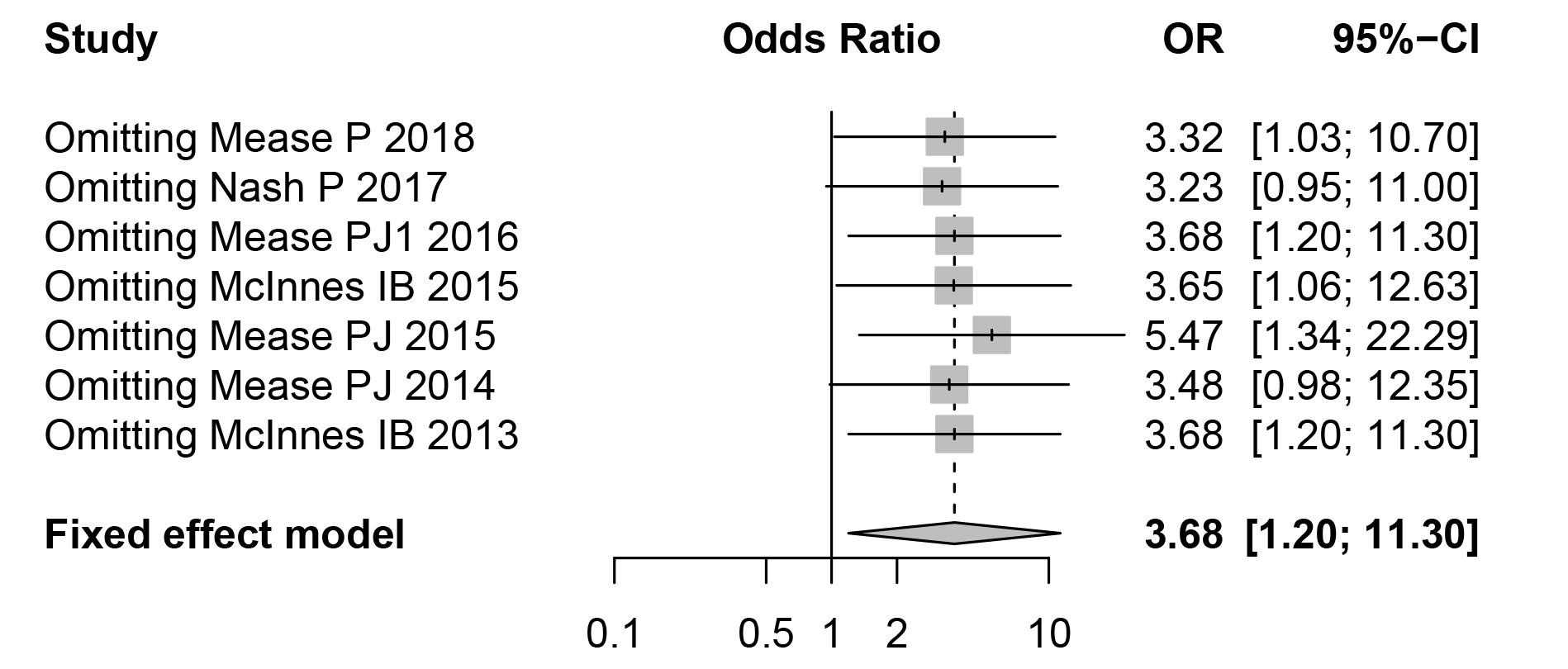

Supplement: Supplementary file 4 [file Image4.TIF]

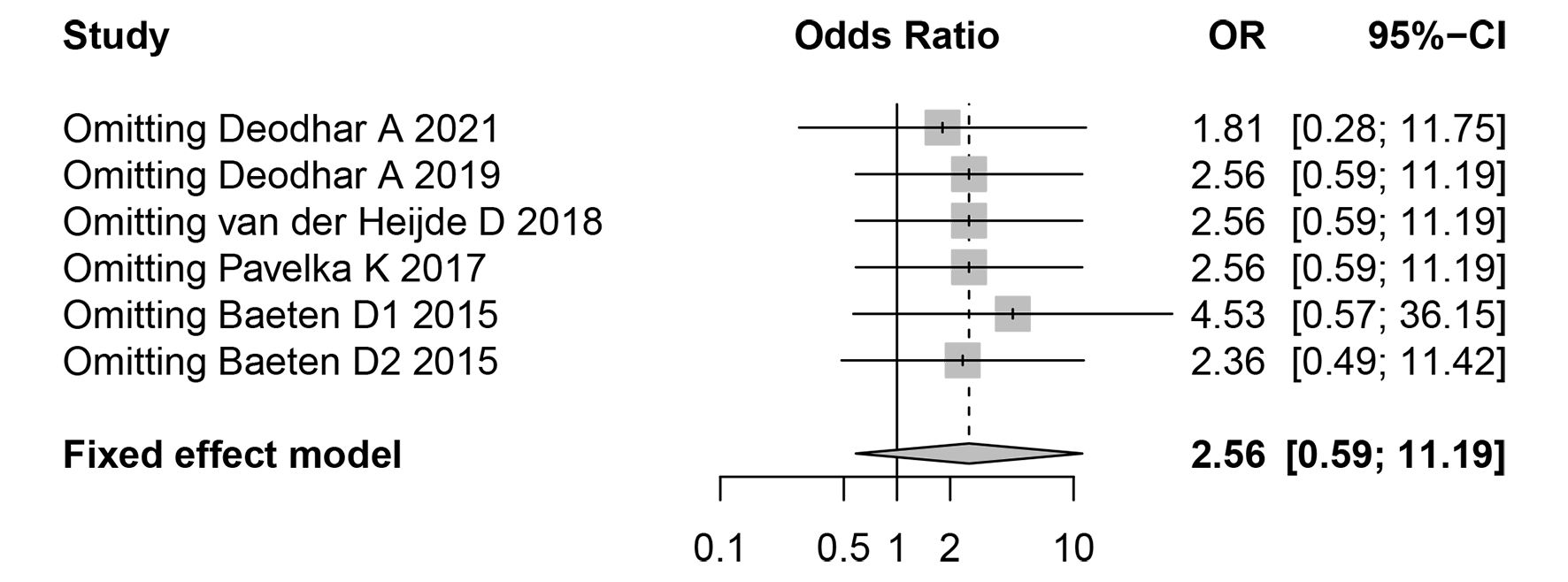

Supplement: Supplementary file 5 [file Image9.TIF]

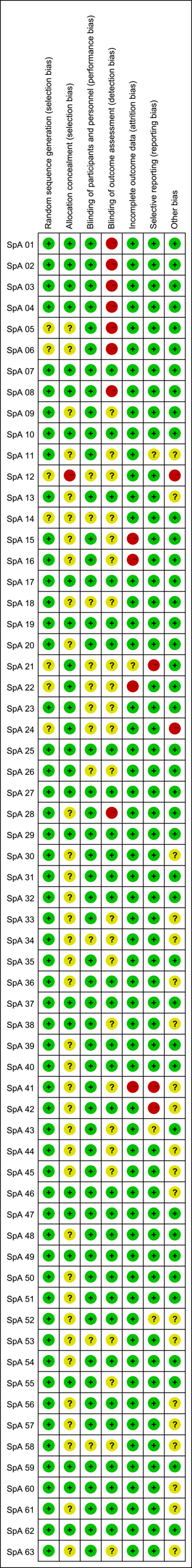

Supplement: Supplementary file 6 [file Image2.TIF]

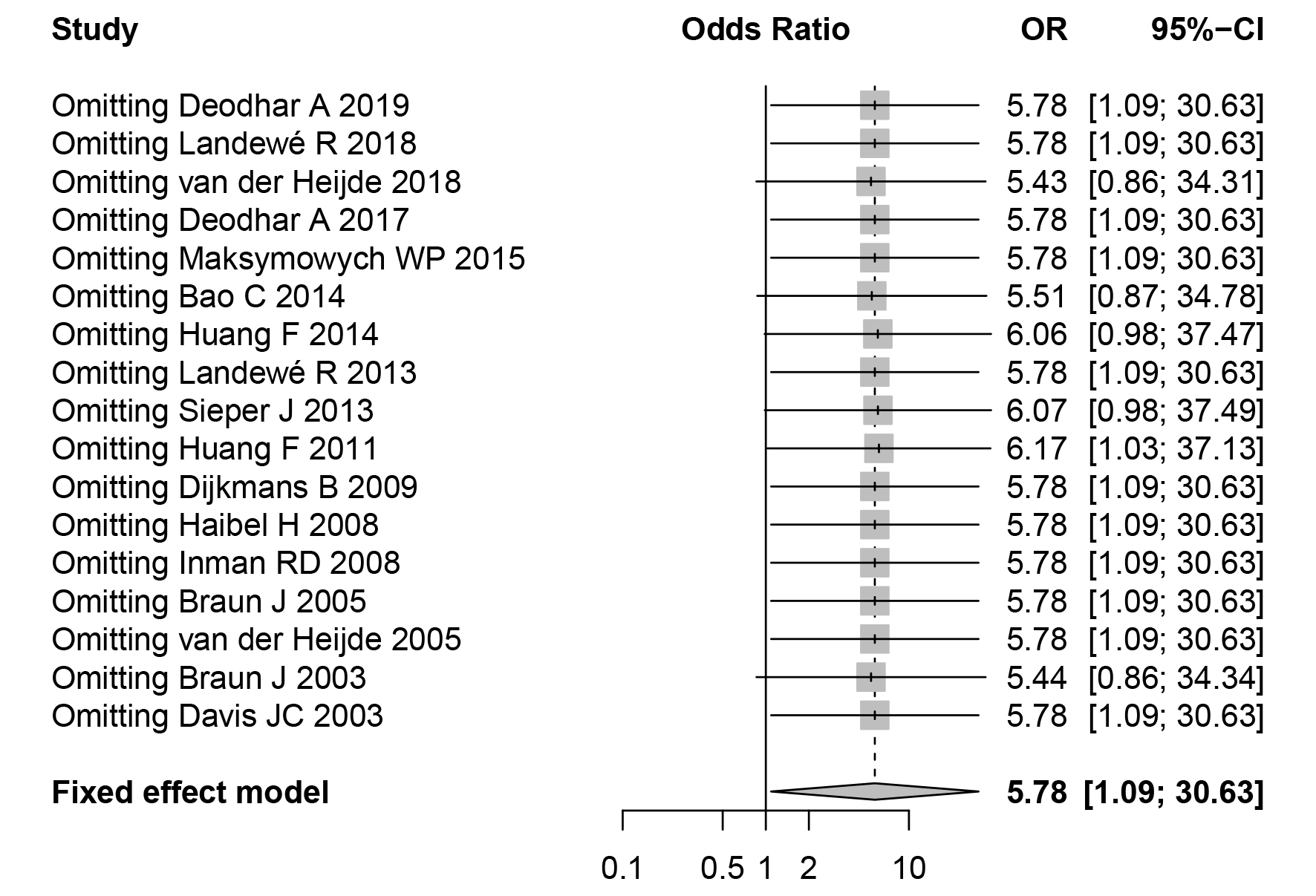

Supplement: Supplementary file 7 [file Image13.TIF]

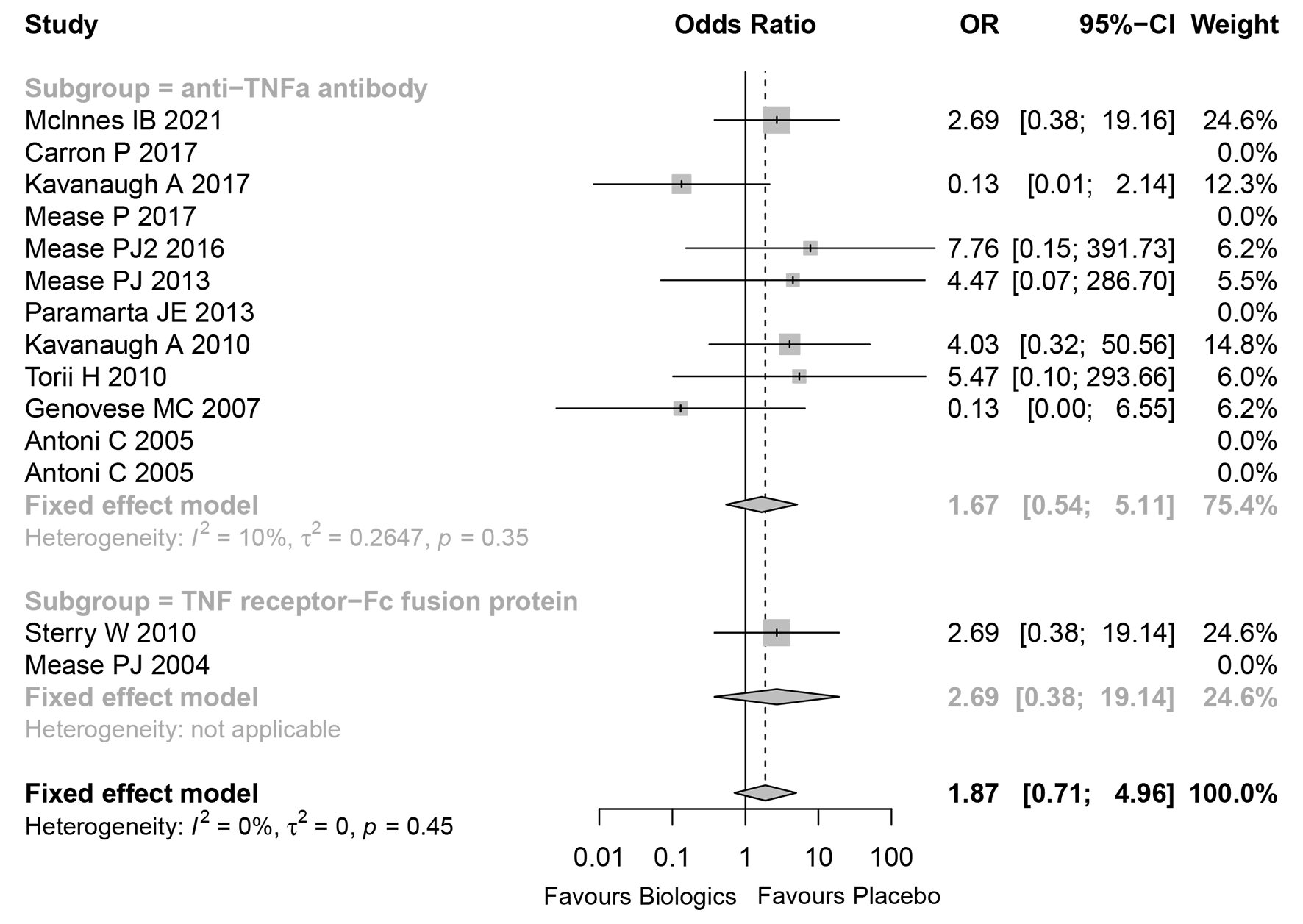

Supplement: Supplementary file 8 [file Image11.TIF]

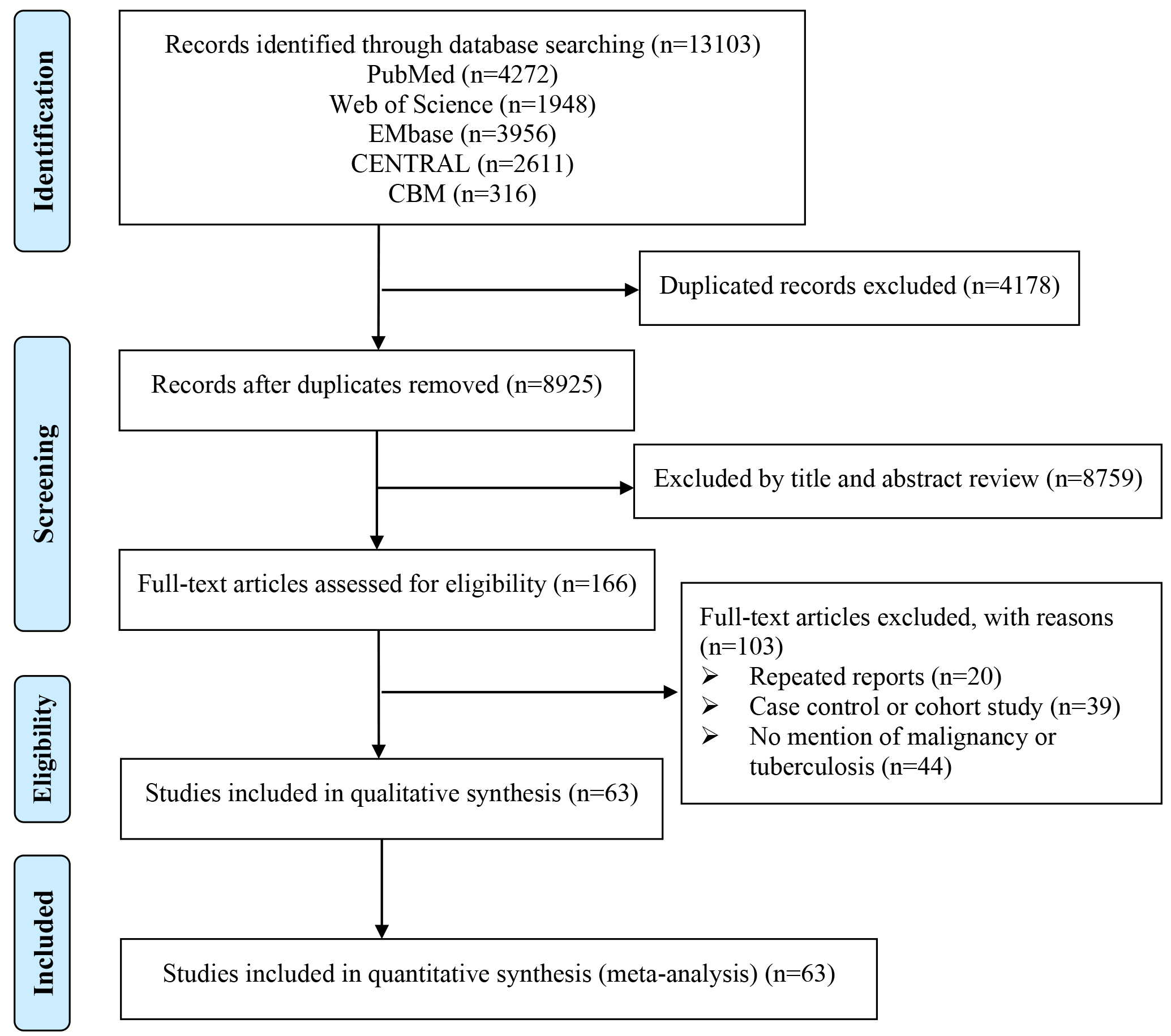

Supplement: Supplementary file 9 [file Image1.TIF]

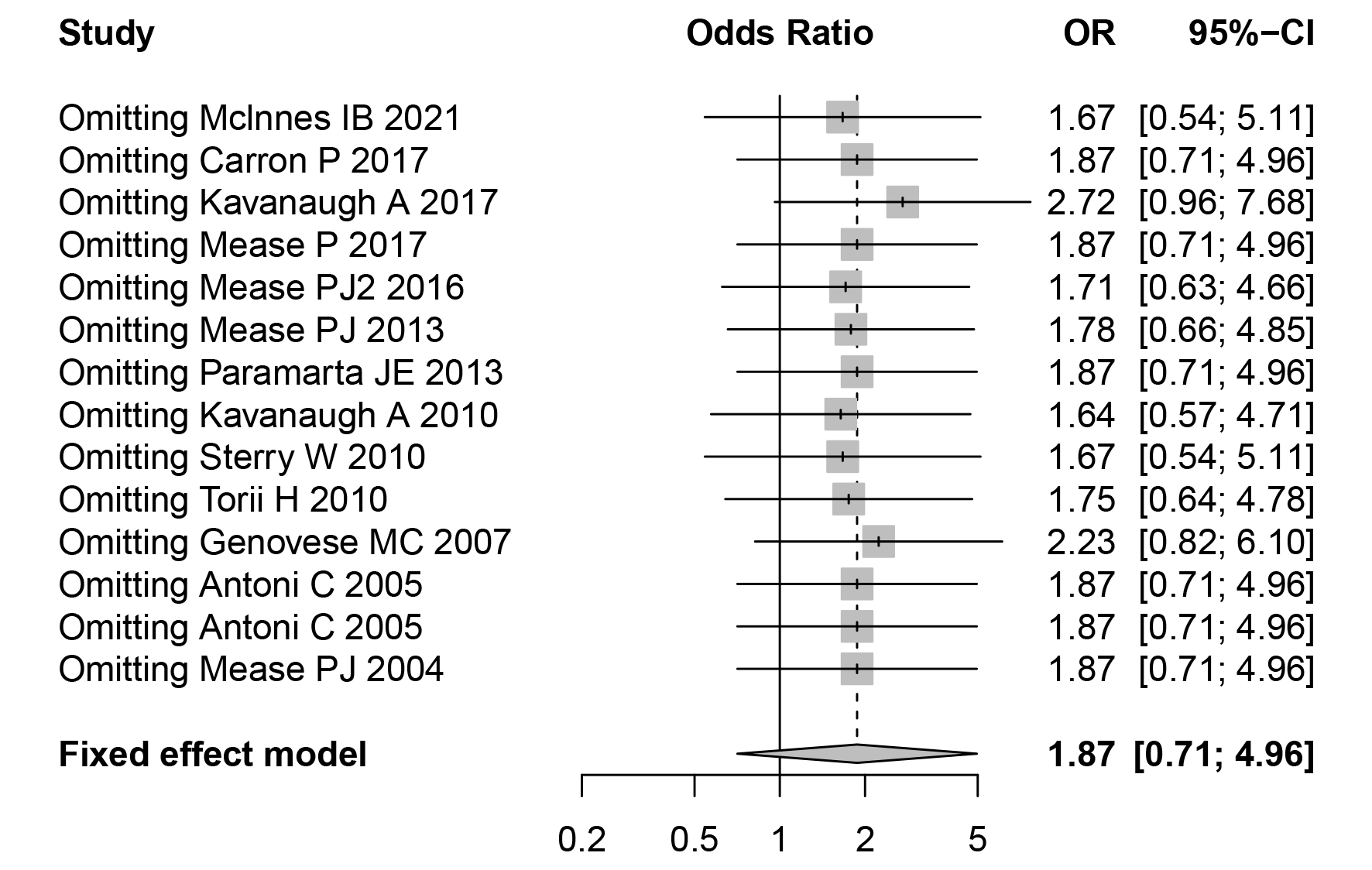

Supplement: Supplementary file 10 [file Image10.TIF]

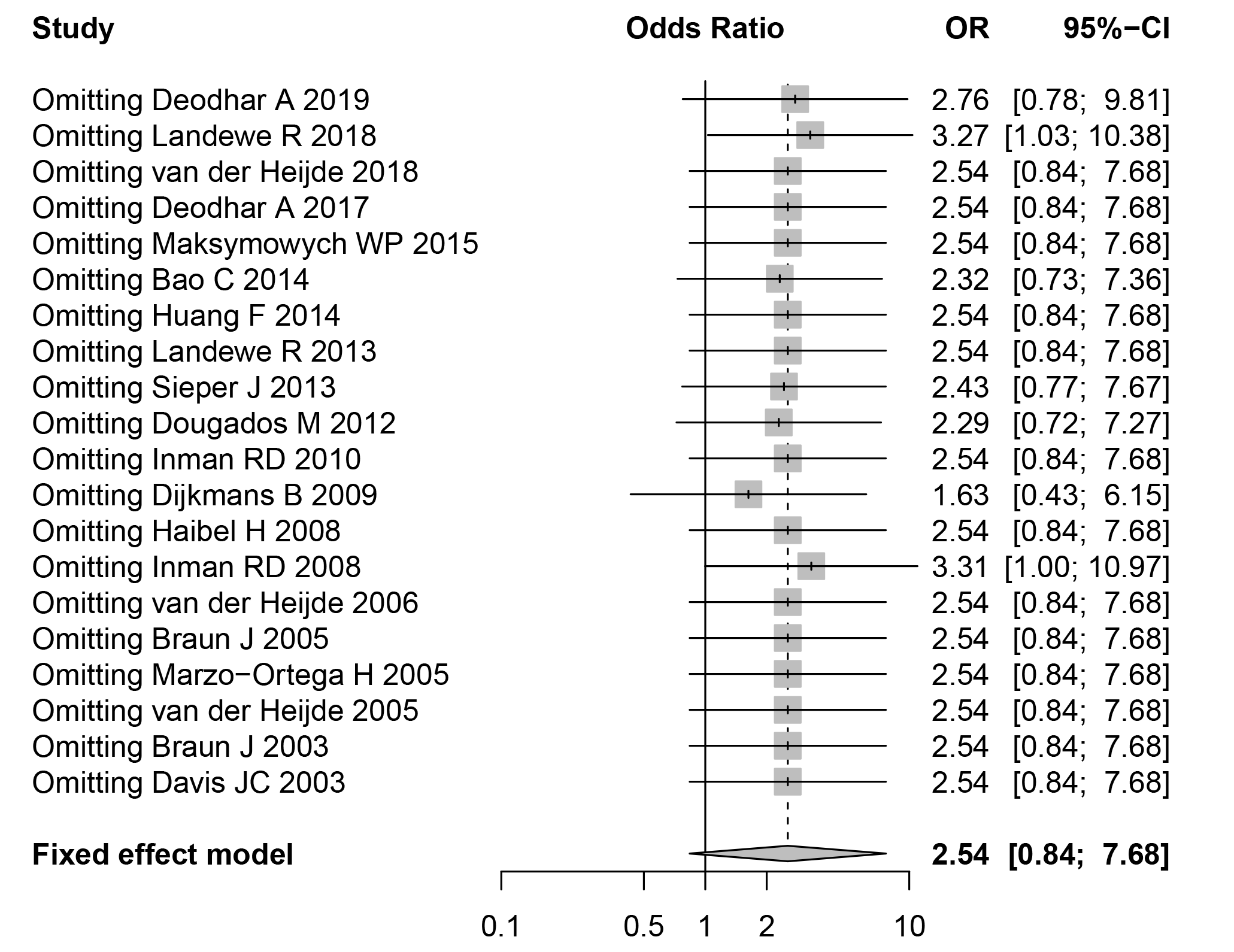

Supplement: Supplementary file 11 [file Image7.TIF]

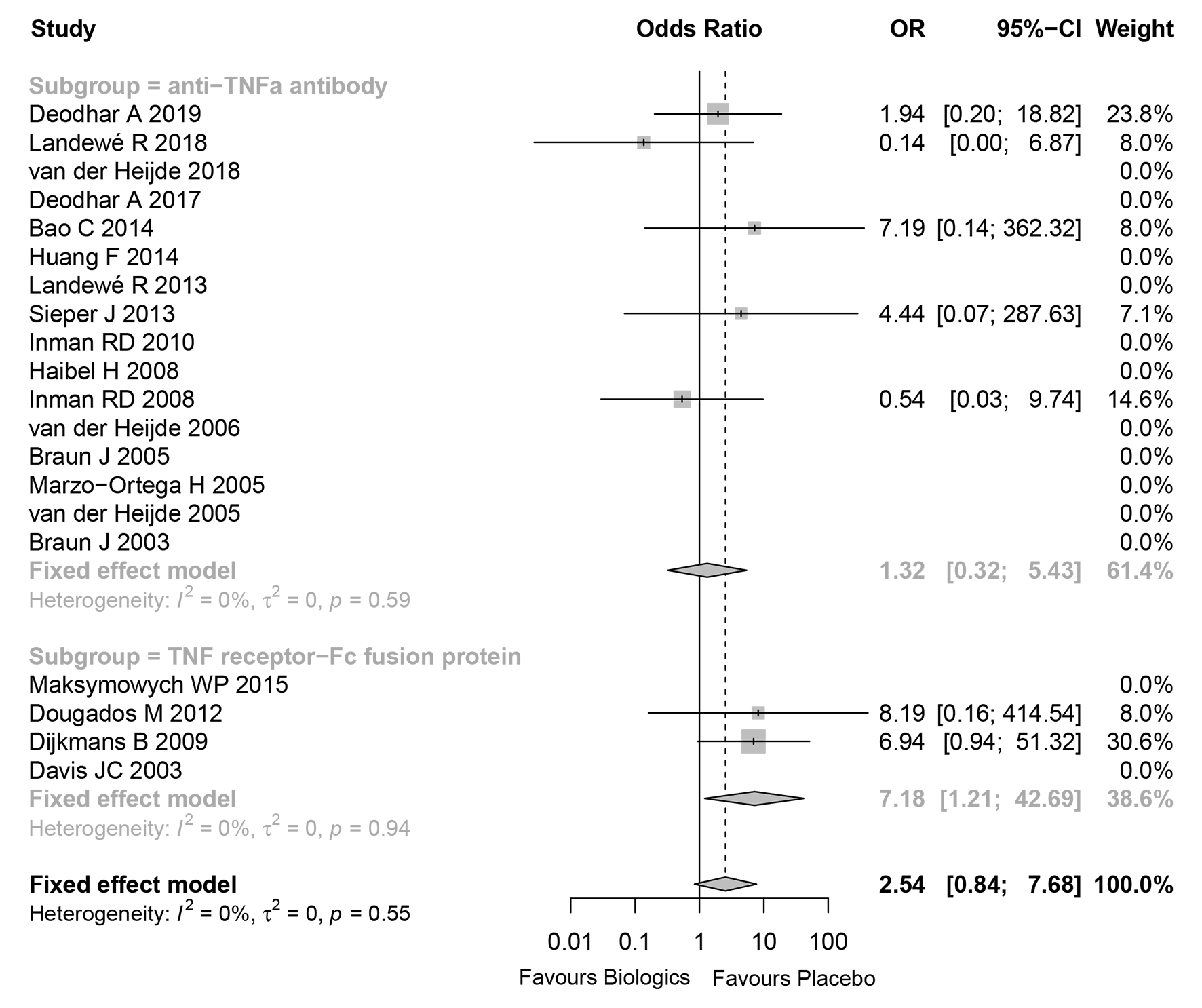

Supplement: Supplementary file 14 [file Image8.TIF]

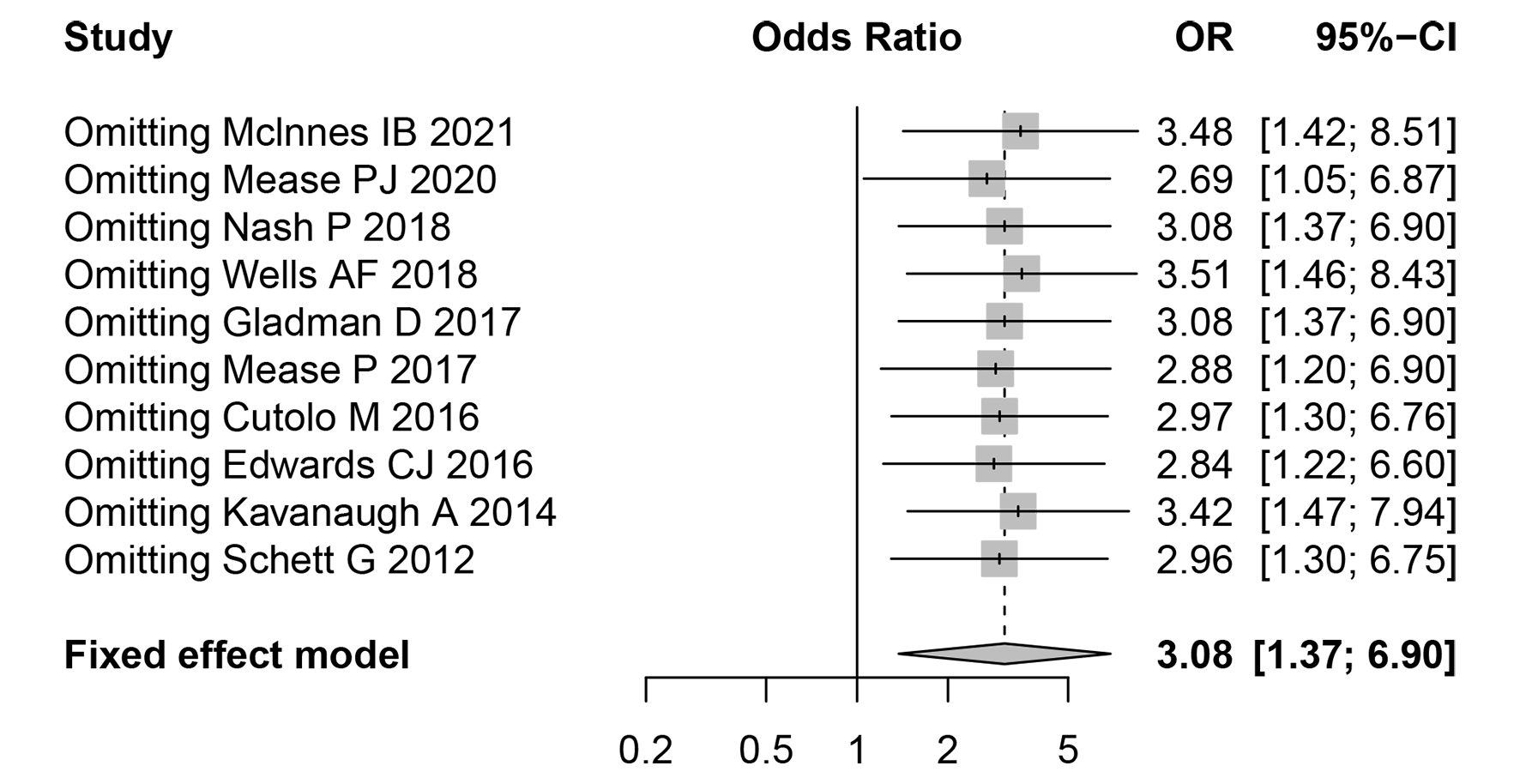

Supplement: Supplementary file 15 [file Image5.TIF]

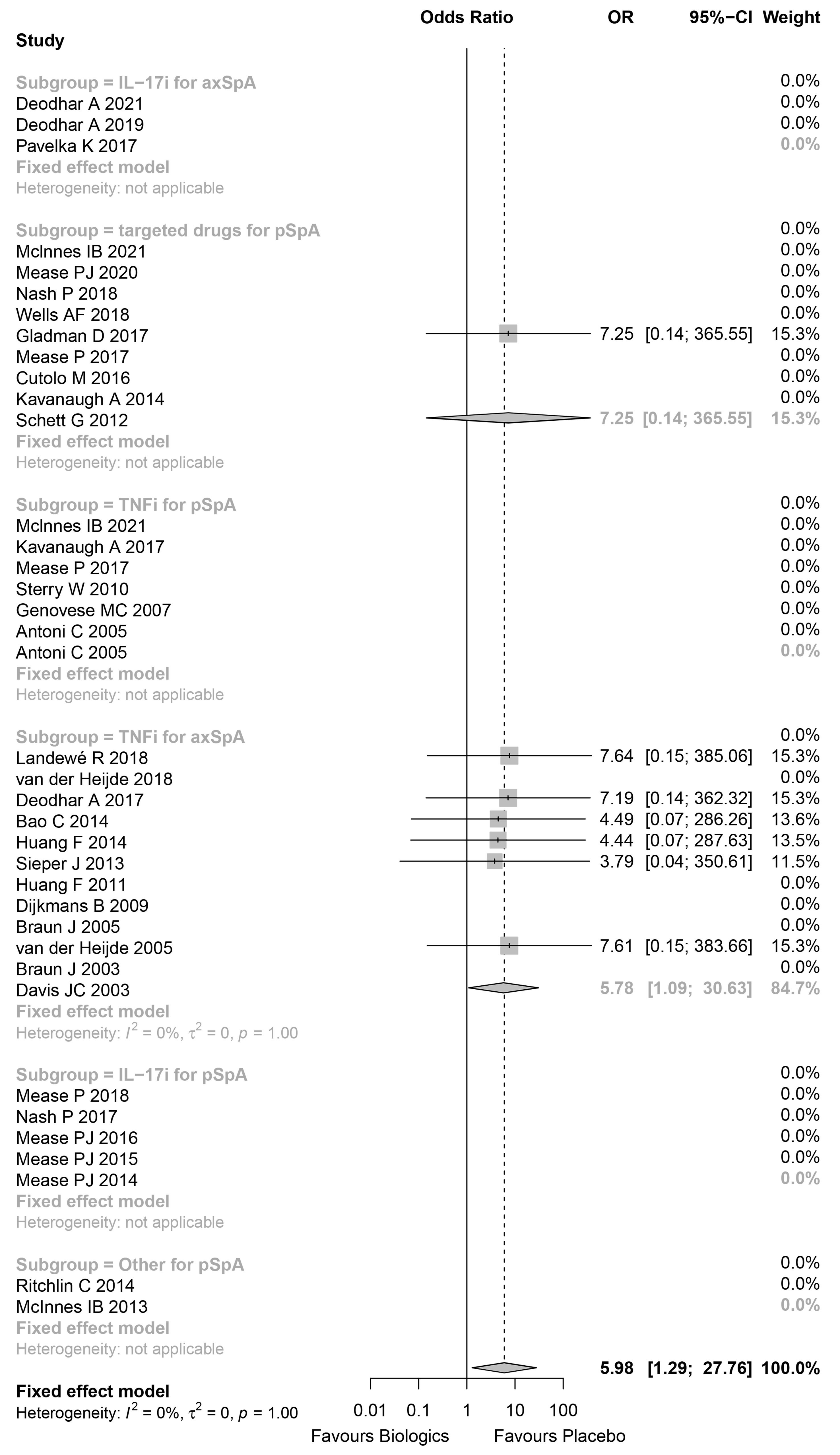

Supplement: Supplementary file 16 [file Image15.TIF]

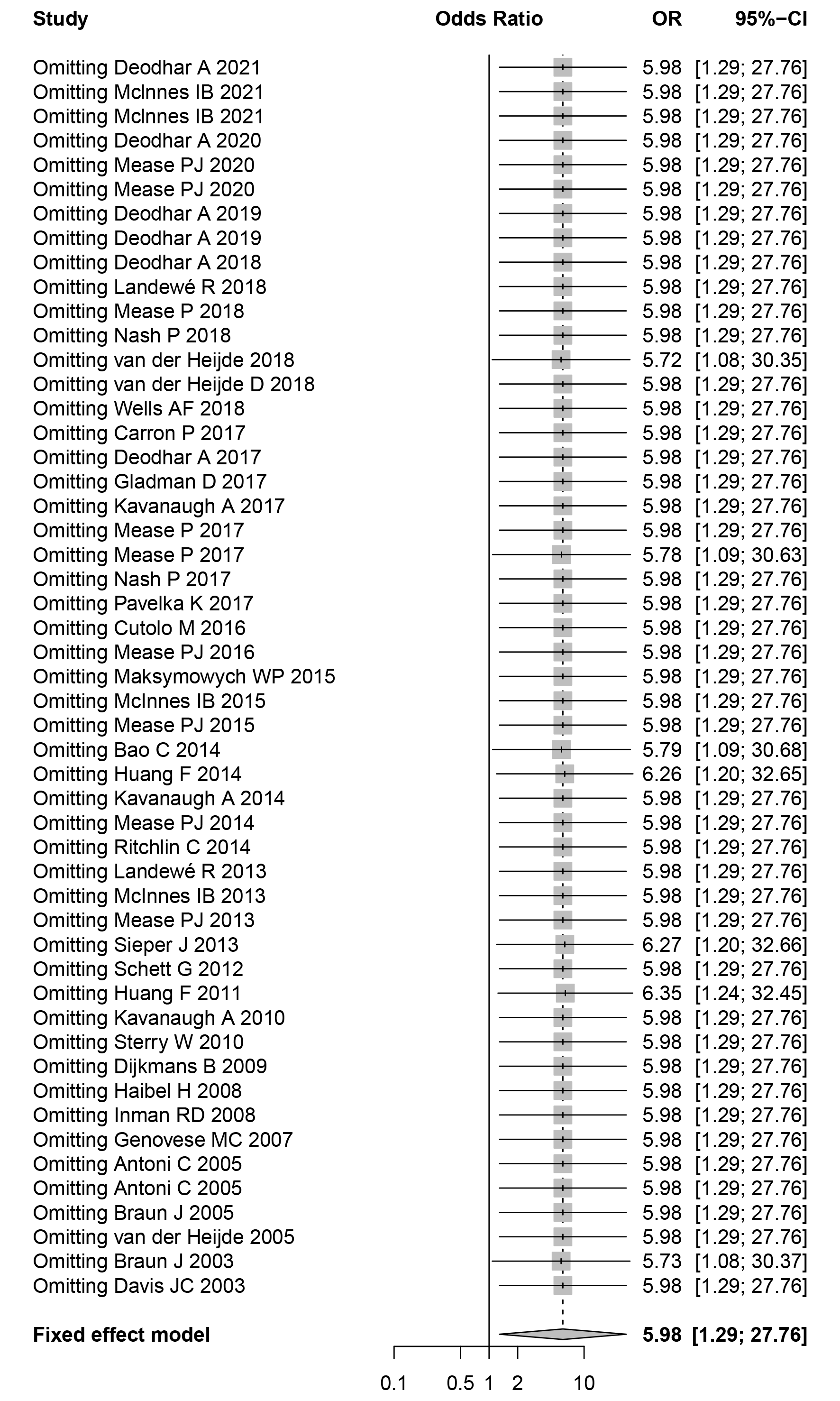

Supplement: Supplementary file 17 [file Image12.TIF]
