# Supplementary material for: Risk of Malignancy and Tuberculosis of Biological and Targeted Drug in Patients With Spondyloarthritis: Systematic Review and Meta-analysis of Randomized Controlled Trials
Source: Front Pharmacol. 2021 Oct 29;12:705669. doi: 10.3389/fphar.2021.705669 (PMC8585981; doi:10.3389/fphar.2021.705669)
Supplement: Supplementary file 18 [file DataSheet1.docx]

**Appendix 1. The details of search strategies for electronic database**

The relevant literature was found by performing following search in PubMed:

#1 "Spondylarthropathies"[Mesh]

#2 "Spondylarthritis"[ Mesh]

#3 "Spondylitis, Ankylosing"[Mesh]

#4 Spondyloar*[Title/Abstract]

#5 spondylar*[Title/Abstract]

#6 "ankylosing spondylitis"[Title/Abstract]

#7 "Psoriatic arthritis"[Title/Abstract]

#8 #1 OR #2 OR #3 OR #4 OR #5 OR #6 OR #7

#9 "immunoglobulin g"[ Title/Abstract]

#10 Monokines [Title/Abstract]

#11 interleukin-6 [Title/Abstract]

#12 "polyethylene glycol"[Title/Abstract]

#13 t-lymphocytes [Title/Abstract]

#14 Infliximab [Title/Abstract]

#15 remicade [Title/Abstract]

#16 adalimumab [Title/Abstract]

#17 humira [Title/Abstract]

#18 trudexa [Title/Abstract]

#19 abatacept [Title/Abstract]

#20 orencia [Title/Abstract]

#21 anakinra [Title/Abstract]

#22 kineret [Title/Abstract]

#23 Certolizumab [Title/Abstract]

#24 Etanercept [Title/Abstract]

#25 Enbrel [Title/Abstract]

#26 Golimumab [Title/Abstract]

#27 simponi [Title/Abstract]

#28 rituximab [Title/Abstract]

#29 Tocilizumab [Title/Abstract]

#30 secukinumab [Title/Abstract]

#31 guselkumab [Title/Abstract]

#32 ustekinumab [Title/Abstract]

#33 ixekizumab [Title/Abstract]

#34 belimumab [Title/Abstract]

#35 brodalumab [Title/Abstract]

#36 bimekizumab [Title/Abstract]

#37 clazakizumab [Title/Abstract]

#38 efalizumab [Title/Abstract]

#39 risankizumab [Title/Abstract]

#40 Upadacitinib [Title/Abstract]

#41 Tofacitinib [Title/Abstract]

#42 #9 OR #10 OR #11 OR #12 OR #13 OR #14 OR #15 OR #16 OR #17 OR #18 OR #19 OR #20 OR #21 OR #22 OR #23 OR #24 OR #25 OR #26 OR #27 OR #28 OR #29 OR #30 OR #31 OR #32 OR #33 OR #34 OR #35 OR #36 OR #37 OR #38 OR #39 OR #40 OR #41

#43 #8 AND #42

All terms were searched as both text words and key words in title and abstract.

The search strategy was adjusted for the searches in EMbase, Web of Science, and CENTRAL.

The relevant literature was found by performing following search in CBM:

#1 "脊柱炎, 强直性"[不加权:扩展]

#2 "强直性脊柱炎"[标题/摘要:智能]

#3 "脊柱关节炎"[标题/摘要:智能]

#4 "银屑病关节炎"[标题/摘要:智能]

#5 #1 OR #2 OR #3 OR #4

#6 "生物因子"[不加权:扩展]

#7 "阿达木单抗"[标题/摘要:智能]

#8 "英夫利昔单抗"[标题/摘要:智能]

#9 "戈利木单抗"[标题/摘要:智能]

#10 "依那西普"[标题/摘要:智能]

#11 "益赛普"[标题/摘要:智能]

#12 "类克"[标题/摘要:智能]

#13 "强克"[标题/摘要:智能]

#14 "赛妥珠单抗"[标题/摘要:智能]

#15 "克拉扎珠单抗"[标题/摘要:智能]

#16 "古塞库单抗"[标题/摘要:智能]

#17 "依克珠单抗"[标题/摘要:智能]

#18 "布罗达单抗"[标题/摘要:智能]

#19 "乌司奴单抗"[标题/摘要:智能]

#20 "阿巴西普"[标题/摘要:智能]

#21 "阿普斯特"[标题/摘要:智能]

#22 "托法替布"[标题/摘要:智能]

#23 "乌帕替尼"[标题/摘要:智能]

#24 "司库奇优单抗"[标题/摘要:智能]

#25 "TNF-α抑制剂"[标题/摘要:智能]

#26 "IL-17抑制剂"[标题/摘要:智能]

#27 "IL-6抑制剂"[标题/摘要:智能]

#28 "IL-23抑制剂"[标题/摘要:智能]

#29 #6 OR #7 OR #8 OR #9 OR #10 OR #11 OR #12 OR #13 OR #14 OR #15 OR #16 OR #17 OR #18 OR #19 OR #20 OR #21 OR #22 OR #23 OR #24 OR #25 OR #26 OR #27 OR #28

#30 #5 AND #29 AND ("随机对照试验"[文献类型])
